# Antibacterial activity of glycerol monolaurate formulated in petrolatum

**DOI:** 10.1128/spectrum.00613-26

**Published:** 2026-06-16

**Authors:** Patrick M. Schlievert, Angie Castillo-Zhagui, Samuel H. Kilgore, Donald Y. M. Leung

**Affiliations:** 1 Department of Microbiology and Immunology, University of Iowa Carver College of Medicine 12243 , Iowa City, Iowa, USA; 2 Department of Pediatrics, National Jewish Health 2930, Denver, Colorado, USA; University of Louisville, Louisville, Kentucky, USA

**Keywords:** *Staphylococcus aureus*, viridans streptococci, lactobacilli, glycerol monolaurate, petrolatum, atopic dermatitis, diabetic ulcers, food allergies

## Abstract

Glycerol monolaurate (GML) is a fatty acid monoester of glycerol that is approved by the FDA as a food additive and component of cosmetics. We previously showed that 50,000 µg/mL of GML solubilized in the non-aqueous K-Y Warming Gel is broadly antibacterial, including *S. aureus* and various streptococcal species, and selectively increases beneficial lactobacilli. GML is also anti-inflammatory, reducing harmful inflammation. In the current study, we examined GML and longer or shorter fatty acid monoesters for their anti-staphylococcal activity. GML had the greatest anti-staphylococcal activity. We then showed *in vitro* that GML solubilized in petrolatum, a non-aqueous gel that has fewer unwanted side effects than K-Y Warming Gel, had enhanced activity in the killing of both methicillin-sensitive and methicillin-resistant *S. aureus*. GML in petrolatum also killed two coagulase-negative staphylococci. Collectively, all staphylococci were killed by GML concentrations of ≥500 µg/mL. This concentration is 100 times below the concentration of GML we previously used *in vivo* in humans as solubilized in K-Y Warming Gel. GML in petrolatum also effectively killed four viridans streptococci, organisms increasingly recognized as pathogenic in atopic dermatitis in children. GML (50,000 µg/mL) in petrolatum was bactericidal for *S. aureus* as tested in a dermatitis model in rabbits. The data suggest that GML in petrolatum, upon additional testing, may become useful in topical management of nasal and skin colonization of children, as well as older individuals, with *S. aureus* and viridans streptococci, and for use in management of Gram-positive bacteria in atopic dermatitis and diabetic ulcers.

**IMPORTANCE:**

Most staphylococcal and streptococcal infections in humans originate from mucous membranes, usually the anterior nares, or from the skin. Glycerol monolaurate (GML) solubilized in petrolatum exhibited potent anti-Gram-positive bacteria activity as tested both *in vitro* and in a staphylococcal rabbit skin model. Because GML is generally recognized as safe (GRAS by FDA) and petrolatum is already approved for topical human use, our data suggest that upon additional testing GML in petrolatum may be effective for topical use in management of nasal and skin colonization with *S. aureus* and viridans streptococci in young children (as well as older individuals), and for use in management of bacterial infections in atopic dermatitis and diabetic ulcers.

## INTRODUCTION

Glycerol monolaurate (GML) is a fatty acid monoester of glycerol and the 12-carbon fatty acid lauric acid. GML is generally recognized as safe (GRAS) by the Food and Drug Administration as a food additive and for use in cosmetics. GML is potently antimicrobial, killing Gram-positive bacteria, Gram-negative bacteria with a lipo-oligosaccharide outer membrane instead of full lipopolysaccharide, enveloped viruses, and fungi (1–5). GML solubilized in K-Y Warming Gel has been used to decolonize the anterior nares of *S. aureus*, a common source of the organism on the skin of patients (6). Further, a clinical study has shown that human breast milk can be used to reduce the symptoms of atopic dermatitis (AD) in children (7); human breast milk is one of only two sites in humans (8, 9) that contain GML, where it is strongly antimicrobial (8). The second site of GML presence in humans is within macrophages stimulated by certain intracellular pathogenic bacteria (9). The mechanism of antibacterial activity of GML correlates with its ability to embed into bacterial membranes and dissipate potential differences across the membranes (3), a property shared with a natural analog, reutericyclin, produced by many lactobacilli (10).

GML is also anti-inflammatory, reducing harmful inflammation (11, 12). Many pathogens cause infections in humans, primarily by disruption of mucosal and skin barriers (1, 2, 13). This suggests that the anti-inflammatory activity, in addition to antimicrobial activity, is important in preventing infections.

Our studies have shown that GML is synergistic in antibacterial activity in the presence of the non-aqueous gel, K-Y Warming Gel, compared with GML alone (3). This raises the possibility that GML can be solubilized in other non-aqueous gels with fewer adverse effects than K-Y Warming Gel. For example, some women, who have been treated with 5% GML in K-Y Warming Gel, experience stinging upon initial application, and the warming property of K-Y Warming Gel depends on partial dehydration of the epithelium (14).

Petrolatum appears to be a milder solubilizing agent for GML. This agent is a semi-solid mixture of hydrocarbons, such as mineral oils and waxes. Petrolatum is commonly used as a mild, but effective, protective skin barrier treatment to prevent water loss. For example, petrolatum is often used on chapped skin associated with rhinorrhea (runny nose) and diaper rash.

AD is a chronic inflammatory condition that mainly affects the skin (15, 16). AD is characterized by chronic itching, scratching and consequent rash development. AD is one of the most common skin conditions and is present in approximately 204 million people globally. Within the U.S., approximately 32 million people have AD. The onset of AD begins most often in children who are between the ages of 2 months to 5 years (17, 18). Additionally, an AD diagnosis may be one of the first steps in a process some investigators refer to as the “atopic march” (18–20). The “atopic march” often begins with AD, followed by food allergy, allergic rhinitis, and eventually asthma. AD may become less severe as a child ages and enters adolescence. However, there are many cases of AD in adulthood.

Patients with AD are susceptible to colonization and infection by Gram-positive bacteria, notably *Staphylococcus aureus* (16). AD in very young children is increasingly being associated with viridans streptococci (21). *S. aureus* is present on the skin of 17% of adults, and for those with AD, the percentage becomes 100% (16). Viridans streptococci are commonly present on the skin of very young children (21). While *S. aureus* may reside naturally in the human flora, most often originating from the anterior nares, patients with AD are infected on their skin by *S. aureus* (16). *S. aureus*, and its secreted toxins, play key roles in driving the symptoms of AD (16, 22, 23), including intense itchiness, and inflammation within skin lesions. For very young children, viridans streptococci may function similarly to *S. aureus*, though these organisms lack many of the potent exotoxins of *S. aureus*. For example, viridans streptococci lack known superantigens, cytotoxins, and lipases. However, they produce potent proteases similar to *S. aureus*. Proteases have been suggested to contribute to the intense itching in AD (22).

There are many other types of *S. aureus* infections. *S. aureus* skin infections are common also (i) in otherwise healthy persons in the form of soft tissue skin infections and (ii) skin infections and diabetic ulcers in association with diabetes mellitus types 1 and 2 (>30 million persons yearly in the United States) (15, 24–26). In hospitals, clonal group USA100 strains, methicillin-sensitive *S. aureus* (MSSA), and methicillin-resistant *S. aureus* (MRSA) are common. USA200 strains, all of which produce the superantigen toxic shock syndrome toxin-1 (TSST-1), are 95% of the time restricted to mucosal surfaces unless the patient has damaged skin (15). The remaining 5% produce wild-type amounts of the cytotoxin α-toxin and can directly infect intact skin. USA300 and USA400 strains are also common (27–29), though USA300 strains are becoming less common; *S. aureus* strains cycle through communities and hospitals in roughly 10-year intervals (30). Both of these latter clonal groups (USA300 and USA400) are typically skin-associated strains, that are mostly MRSA, causing impressive skin infections. USA100-400 strains may spread from skin or mucosal surfaces to cause highly fatal hemorrhagic pneumonia. Other USA clonal groups (USA500–USA1100) can also be associated with skin infections (31).

*S. aureus* skin infections in diabetes mellitus patients are exceptionally common (15, 25). Obesity and pre-diabetes mellitus type 2 increase *S. aureus* skin infections, approaching 100% when people develop diabetes 2 (25). We have evaluated nine people with diabetes mellitus type 2 for the presence of *S. aureus* (25). Based on swabbing their palm, forearm, and axillary skin surfaces, these people have 10^11^–10^13^*S. aureus* on their total skin surfaces, or up to 1 cubic inch. The isolated *S. aureus* were MRSA and MSSA and produced the superantigens enterotoxin C (SEC) and toxic shock syndrome toxin-1 (TSST-1). Two of the patients ultimately succumbed to septic infections due to the same *S. aureus* that colonized their skin. Also, we and others have shown that the superantigens of *S. aureus* have the ability to induce diabetes mellitus type two changes in experimental animals and human adipocytes (25, 26, 32). Diabetes mellitus-associated foot ulcers are severe complications of diabetes (33). These lesions are exceptionally difficult to heal, and they easily become infected with *S. aureus*, leading to amputations in patients with uncontrolled diabetes.

This study was undertaken to examine the ability of GML alone and GML solubilized in petrolatum to kill various species of staphylococci, including both coagulase-positive *S. aureus* and coagulase-negatives (*S. epidermidis* and *S. capitis*), and viridans streptococci. We show that all staphylococci and viridans streptococci tested were killed by GML. However, in the presence of petrolatum, GML is significantly more active than GML alone. This suggests that GML in petrolatum upon additional testing could be an effective topical therapeutic to help in preventing and managing skin conditions due to these Gram-positive bacteria.

## RESULTS

### Solubilization of GML in petrolatum

Food-grade GML was solubilized in petrolatum (both petrolatum and generic) at GML concentrations as high as 5% (50,000 µg/mL). Both GML and petrolatum were stable at 65°C, the temperature used to solubilize GML. Petrolatum is a semi-solid at room temperature but is a liquid at 65 °C. After solubilization of 5% GML in petrolatum, the mixture could be stored at room temperature without GML crystallizing out of solution. This is different from 5% GML in K-Y Warming Gel where GML begins to crystallize out of solution at room temperature. The 5% amount of GML was chosen for the highest concentration studied since this was the highest concentration of GML used in pilot and clinical trial studies in K-Y Warming Gel as tested in adults, either on vaginal or nasal mucosa (6, 14).

### Anti-*S. aureus* activity of fatty acid monoesters

**Fig 1:**
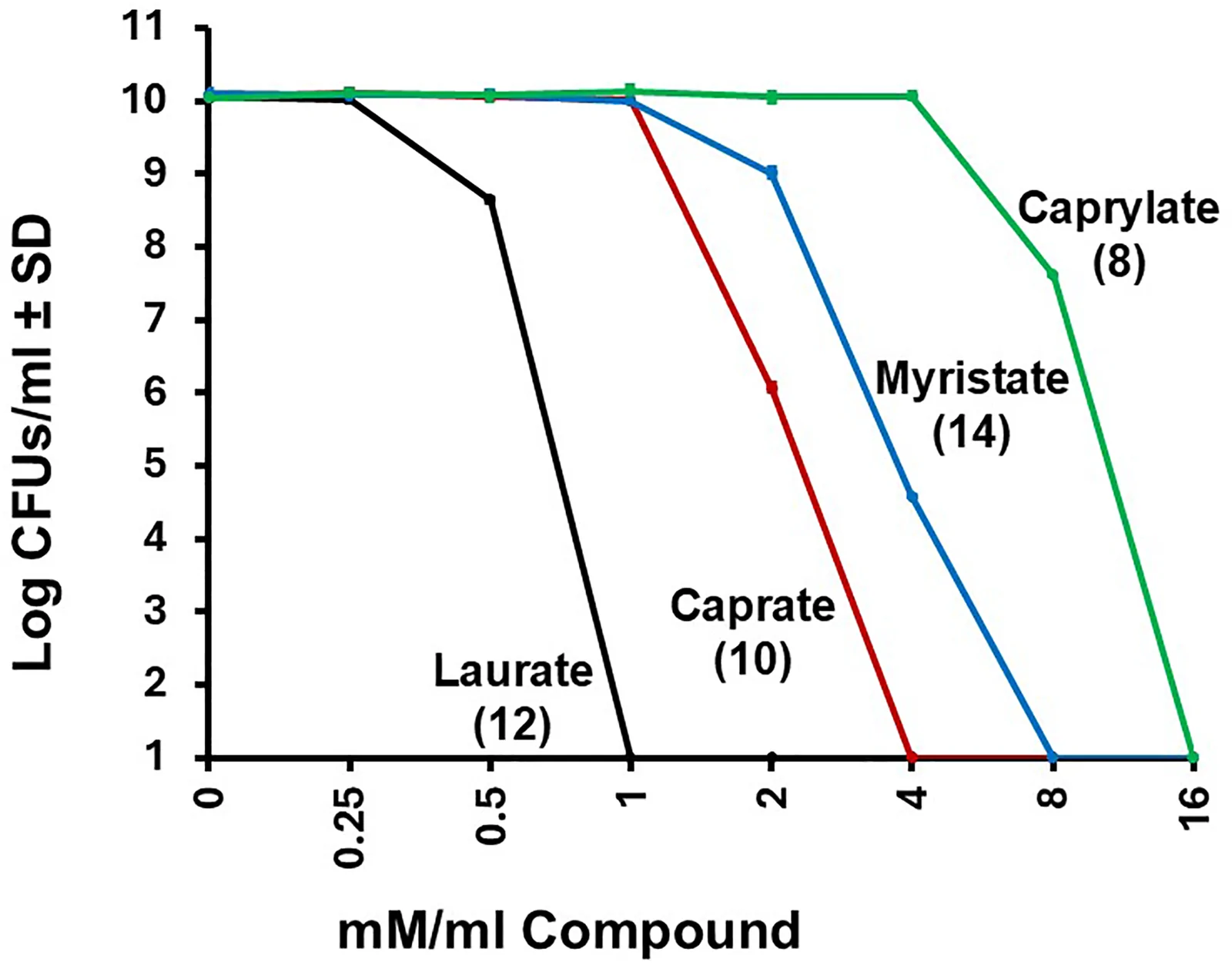
Anti-*S*. *aureus* activity in Todd Hewitt broth of glycerol monoesters (glycerol monolaurate [GML], glycerol monocaprate [caprate], glycerol monomyristate [myristate], and glycerol monocaprylate [caprylate]). Numbers in parentheses are fatty acid side chain lengths of the monoesters. Data are reported as log_10_ colony-forming units (CFUs)/mL ± standard deviation (SD) after 24-h incubation with *S. aureus* MN8 (inoculum size 9.8 × 10^6^/mL).

We have previously published multiple studies of the anti-staphylococcal and anti-streptococcal activity of GML (3, 34, 35). We have not previously assessed other fatty acid monoesters. We thus evaluated GML (12-carbon side chain) compared with glycerol monocaprylate (8-carbon side chain), glycerol monocaprate (10-carbon side chain), and glycerol monomyristate (14-carbon side chain) for ability to kill *S. aureus* after 24-h incubation in Todd Hewitt broth growth medium. Because the molecular weight of each compound differs, the data were reported in mM (Fig. 1). GML was the most anti-staphylococcal with 1 mM yielding no *S. aureus*. The other fatty acid monoesters were also anti-staphylococcal with 4 mM glycerol monocaprylate yielding no *S. aureus*, 8 mM glycerol monomyristate yielding no *S. aureus*, and 16 mM glycerol monocaprate yielding no *S. aureus*. All of our subsequent studies evaluated only GML for activity since it was the most potent against *S. aureus*, and data were reported as micrograms per milliliter required for bactericidal activity. It is also important to note that all *S. aureus* strains produce glycerol ester hydrolase (lipase) with the capability to cleave the tested monoesters (36). The greater GML activity in the presence of *S. aureus* lipases suggests our next studies with GML may be the most important for development of topical-use antimicrobial monoesters.

### Anti-*S. aureus* activity of GML alone, petrolatum alone, and GML + petrolatum

**Fig 2:**
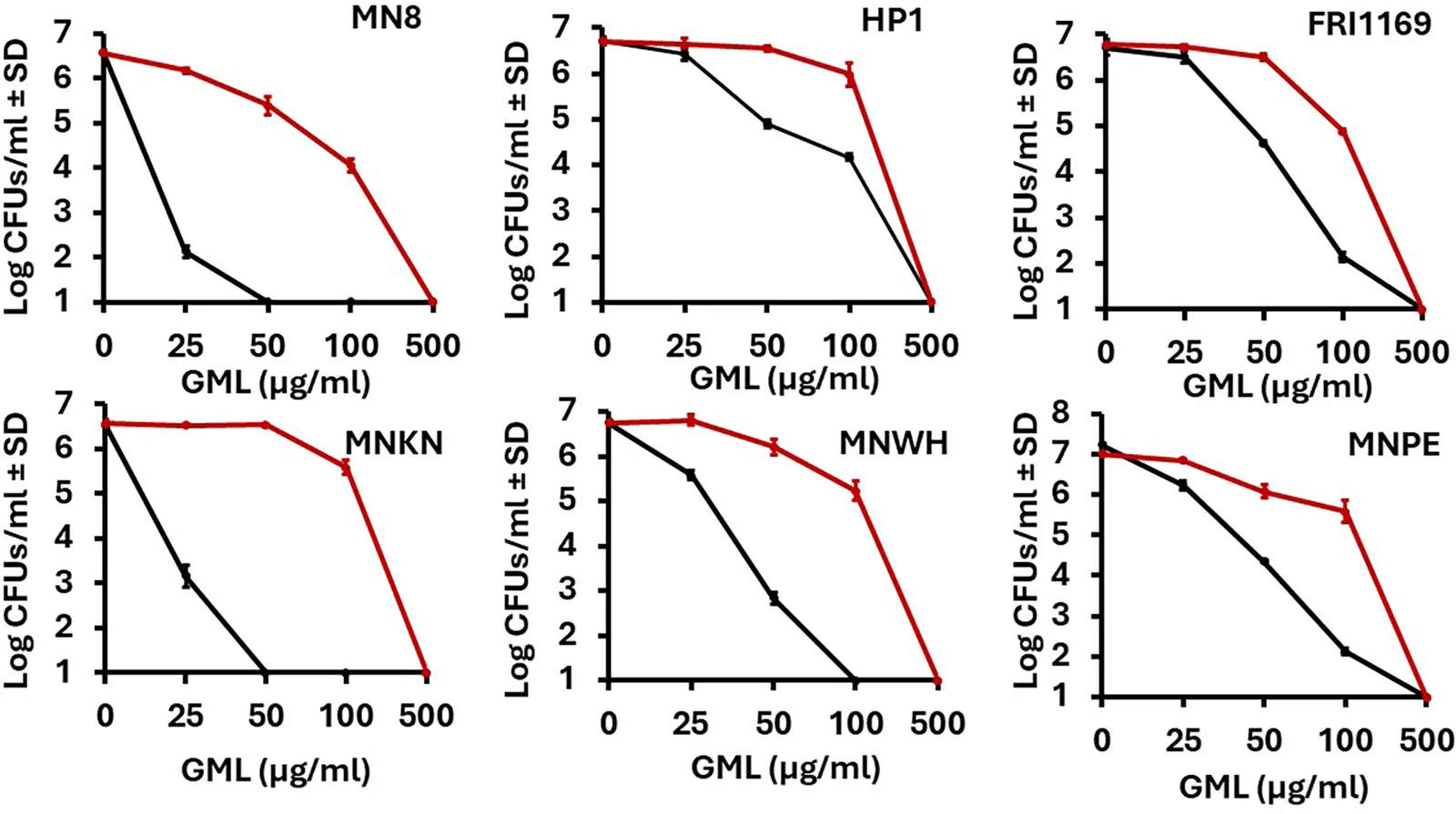
Anti-*S*. *aureus* activity of glycerol monolaurate (GML) in phosphate-buffered saline (GML in PBS; red bars) versus GML in petrolatum (GML in petrolatum; black bars). Data shown are log_10_ colony-forming units (CFUs)/mL ± standard deviations (SDs). Treatment groups list the µg/mL of GML in either phosphate-buffered saline or petrolatum. With use of Student’s *t*-test analysis of unpaired data, the means for GML in petrolatum versus GML in PBS at GML concentrations of 25, 50, and 100 µg/mL were significantly different with *P* < 0.001 for strains MN8, MNKN, MNWH, and MNPE. The means for GML in petrolatum versus GML in PBS at GML concentrations of 50 and 100 µg/mL were significantly different with at *P* < 0.001 for strains HP1 and FRI1169. The other means (GML) were not different (*P* > 0.05).

We previously published that GML alone is bactericidal for toxic shock syndrome (TSS) *S. aureus* strain MN8 (3, 34, 37). This organism is typical of menstrual TSS organisms in that it is positive for TSS toxin-1 (TSST-1), is methicillin-sensitive, and belongs to clonal group USA200 (CC30) (38). GML alone killed *S. aureus* MN8 by >3 log at average concentrations of ≥300 µg/mL (0.03%) in Todd Hewitt growth medium (3, 34, 37). With this as background, we evaluated GML alone, petrolatum alone, and petrolatum + various concentrations of GML (Fig. 2). We evaluated both methicillin-sensitive *S. aureus* (MN8 [USA200], MNPE [USA200], FRI1169 [USA200], HP1 [USA300-related]), and MNKN (USA400) and MNWH (USA200) both methicillin-resistant *S. aureus* (MRSA).

These experiments were performed with GML in petrolatum or PBS, the latter where GML has a solubility limit of 100 µg/mL. However, we previously showed that GML exerts it anti-staphylococcal effect at concentrations well above the solubility limit (3, 34, 37). We assume this results as progressively more GML becomes embedded in the bacterial membrane, and additional GML can then become solubilized. We define bactericidal as the reduction by ≥3 log_10_ CFUs/mL. GML alone in PBS was bactericidal for *S. aureus* only at approximately 500 µg/mL, versus 50 (for some *S. aureus* strains), 100, and 500 µg/mL for GML + petrolatum. Complete killing of *S. aureus* by GML alone in PBS or GML in petrolatum was observed at 500 µg/mL. Petrolatum alone was not antimicrobial for *S. aureus* over the 24-h test period.

**Fig 3:**
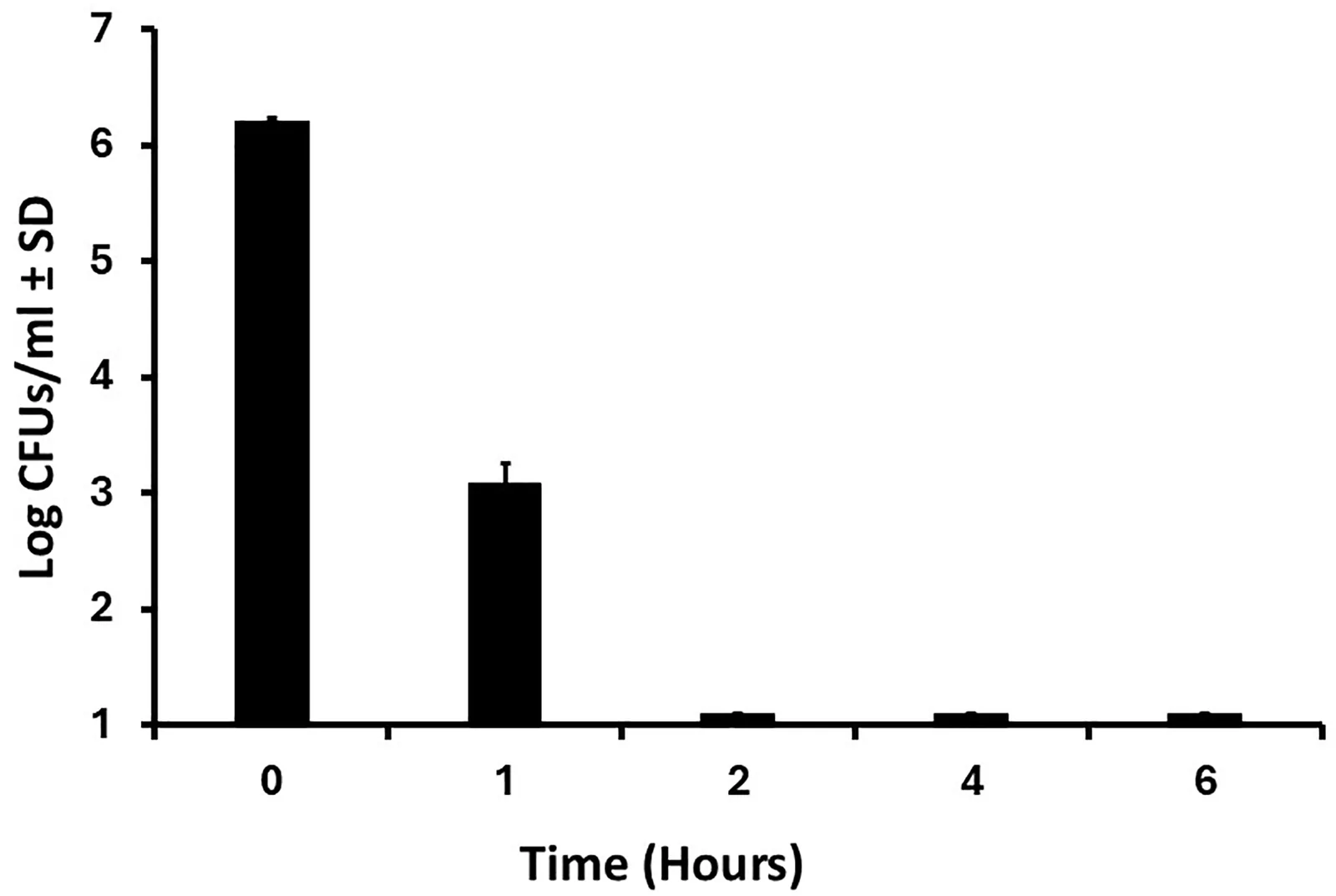
Time-course for killing of *S. aureus* MN8 by GML (500 µg/mL) in petrolatum. SD = standard deviation.

We also performed a time-course experiment for GML in petrolatum to kill *S. aureus* MN8 (Fig. 3). GML in petrolatum was bactericidal by 1 h post-inoculation (*P* < 0.0002) compared with the zero time, with complete killing by 2 h.

**Fig 4:**
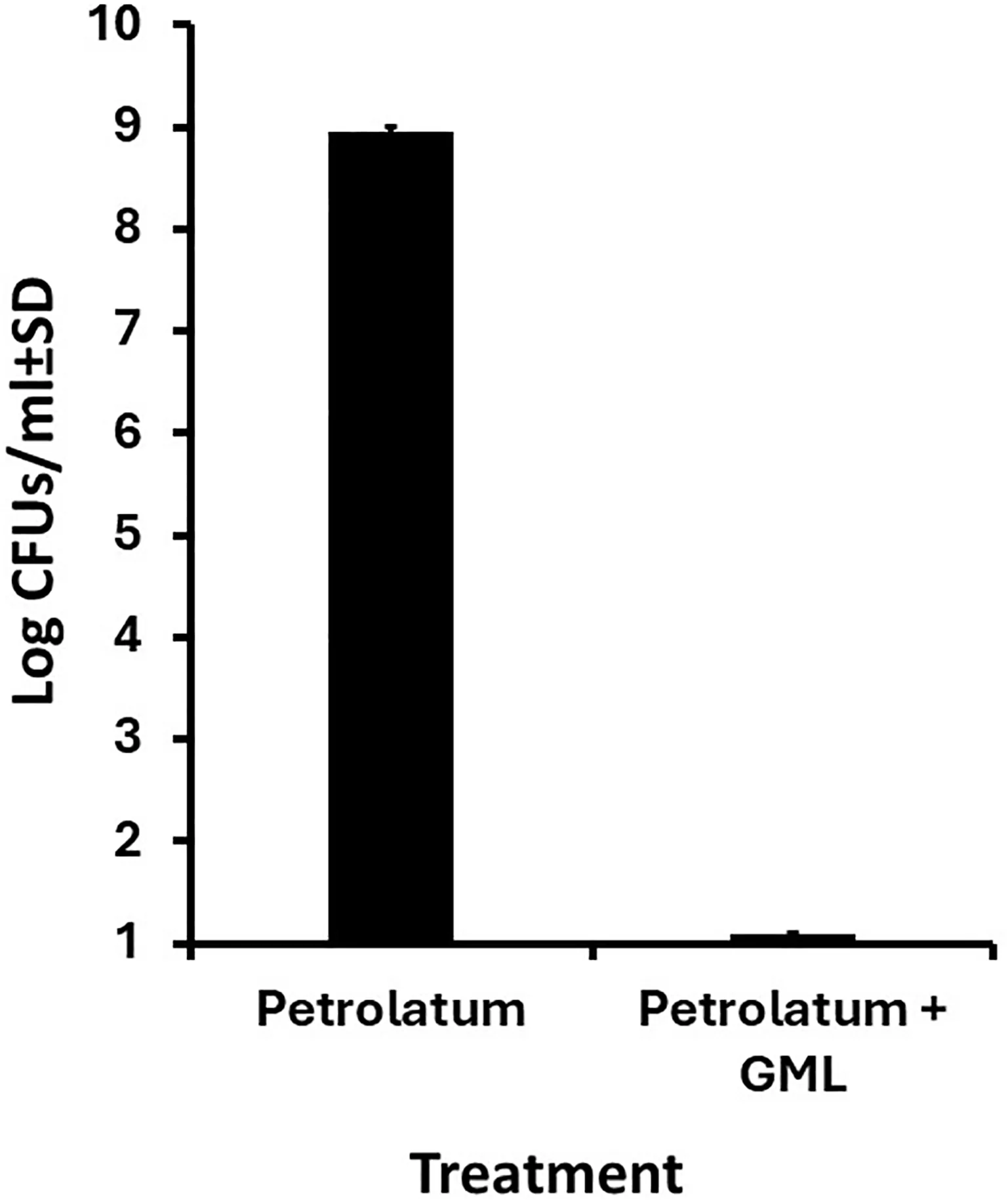
Treatment of New Zealand white rabbits (4/group) on their shaved flanks with either 0.5 mL of petrolatum or 0.5 ml of 50,000 µg/mL of GML (5%) in petrolatum after exposure to approximately 1 × 10^9^ *S. aureus* MN8 in a 0.1 mL volume (2 × 2 cm area). CFUs/mL ± standard deviation (SD) was determined after 8 h. Mean differences between the two groups was *P* < 6.4 × 10^−9^.

We previously showed that 5% (50,000 µg/mL) of GML in K-Y Warming Gel was effective in treating dermatitis in a rabbit model (39). K-Y Warming Gel gets its warming name from pulling moisture out of mucosal tissues, leading to a warm sensation, but at the same time potentially increasing drying. This latter property would be expected to reduce its use with GML, particularly in children. Thus, we evaluated 5% GML (50,000 µg/mL) in petrolatum, compared with petrolatum alone for treating *S. aureus* MN8 dermatitis in rabbits (Fig. 4). Rabbits, 4/group were challenged on flanks with 1 × 10^9^ CFUs/0.1 mL in a 2 × 2 cm area. The flanks were then swabbed with GML + petrolatum, compared with petrolatum alone. CFUs/0.1 mL were determined after 8 h. GML + petrolatum led to complete elimination of *S. aureus* MN8, whereas petrolatum alone led to no reduction in *S. aureus* MN8.

### Effect of GML + petrolatum effect on *Lactobacillus crispatus* and coagulase-negative staphylococci

We have previously shown *in vitro*, *in vivo* vaginally in rhesus macaques, and *in vivo* vaginally in women, that GML (5%, 50,000 µg/mL) +K-Y Warming Gel led to no killing of lactobacilli, and indeed, led to increases in normal microbiome lactobacilli vaginally in women (1, 2, 5, 14, 40). It was proposed that this positive effect resulted from lactobacilli using GML as a quorum growth stimulant. GML is an analog of the quorum growth stimulant present in many lactobacilli, reutericyclin (10). Our studies have shown that GML and reutericyclin have the same spectrum of antimicrobial activity, both without negative impact on lactobacilli (3, 4).

**Fig 5:**
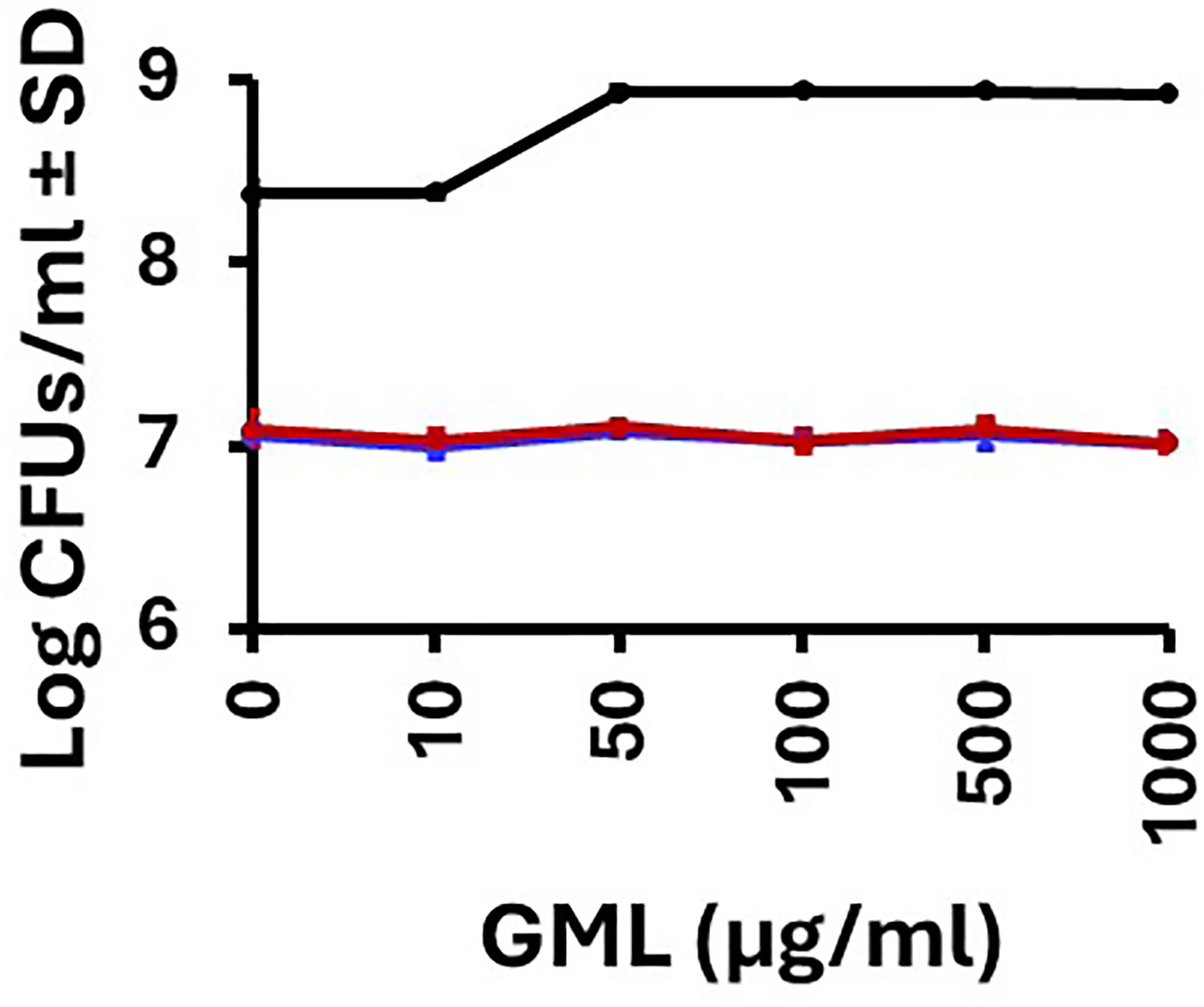
Effect of GML alone in Todd Hewitt and GML + petrolatum versus GML + PBS of viability of *Lactobacillus crispatus*. Incubation of cultures was stationary at 37°C for 24 h. Means ± standard deviations (SDs) are shown. The black line shows the results of growth of *L. crispatus* in Todd Hewitt broth from an inoculum of approximately 10^7^ CFU/mL to the value listed after 24-h incubation. Means of growth of *L. crispatus* in Todd Hewitt at GML concentrations of ≥50 µg/mL were significantly different (Student’s *t*-test) from means at GML concentrations of 0 and 10 µg/mL (*P* < 0.0001). The blue line represents *L. crispatus* in GML + petrolatum. The red line represents *L. crispatus* in GML + PBS. The means of the blue and red lines at all concentrations of GML were not different.

In this study, we evaluated the effect of GML alone on the growth of *L. crispatus* in Todd Hewitt broth, and the effect of GML in petrolatum versus GML in PBS on viability of the same lactobacilli (Fig. 5). GML alone in Todd Hewitt broth did not kill *L. crispatus* at any concentration tested (up to 1,000 µg/mL). The lactobacilli grew to significantly higher 24-h stationary phases at GML concentrations of ≥50 µg/mL, indicating GML was a growth stimulant for this organism (*P* < 0.0001 for all GML concentrations ≥50 µg/mL). GML in petrolatum and GML in PBS did not result in killing of *L. crispatus*. No growth was observed in either condition since culture media were not added.

**Fig 6:**
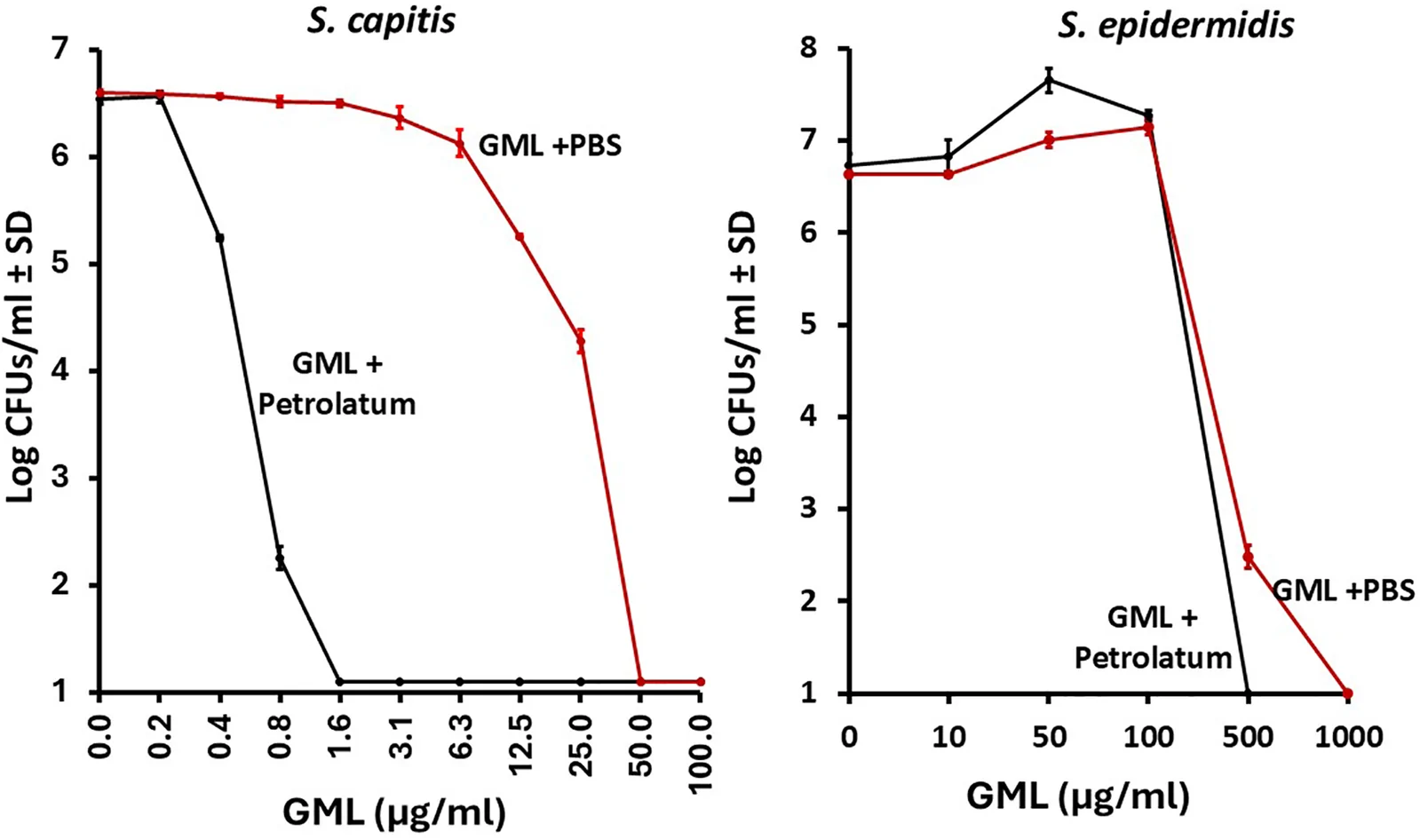
Effect of GML + petrolatum versus GML + PBS on two strains of coagulase-negative staphylococci (*S. capitis* and *S. epidermidis*). Both treatment groups were tested for 24 h at 37°C. Data are log_10_CFU/mL ± SD (standard deviation).

We also tested GML alone in PBS, petrolatum alone, and GML + petrolatum for effect on growth of one strain each of other skin microbiome organisms, *S. capitis* and *S. epidermidis* (Fig. 6). The clinical isolate of *S. capitis* (41) was overall more susceptible to killing by GML than *S. epidermidis*, whether treated with GML in petrolatum or in PBS. GML in petrolatum was bactericidal for *S. capitis* (≥3 log killing) at 0.8 µg/mL, with complete killing at 1.6 µg/mL, compared with 50 µg/mL in PBS, whether bactericidal activity or complete killing was assessed. The normal microbiome *S. epidermidis* was resistant to killing by GML in petrolatum and in PBS until concentrations of GML of 500 or 1,000 µg/mL, respectively.

### Effect of GML + petrolatum effect on viridans streptococci

**Fig 7:**
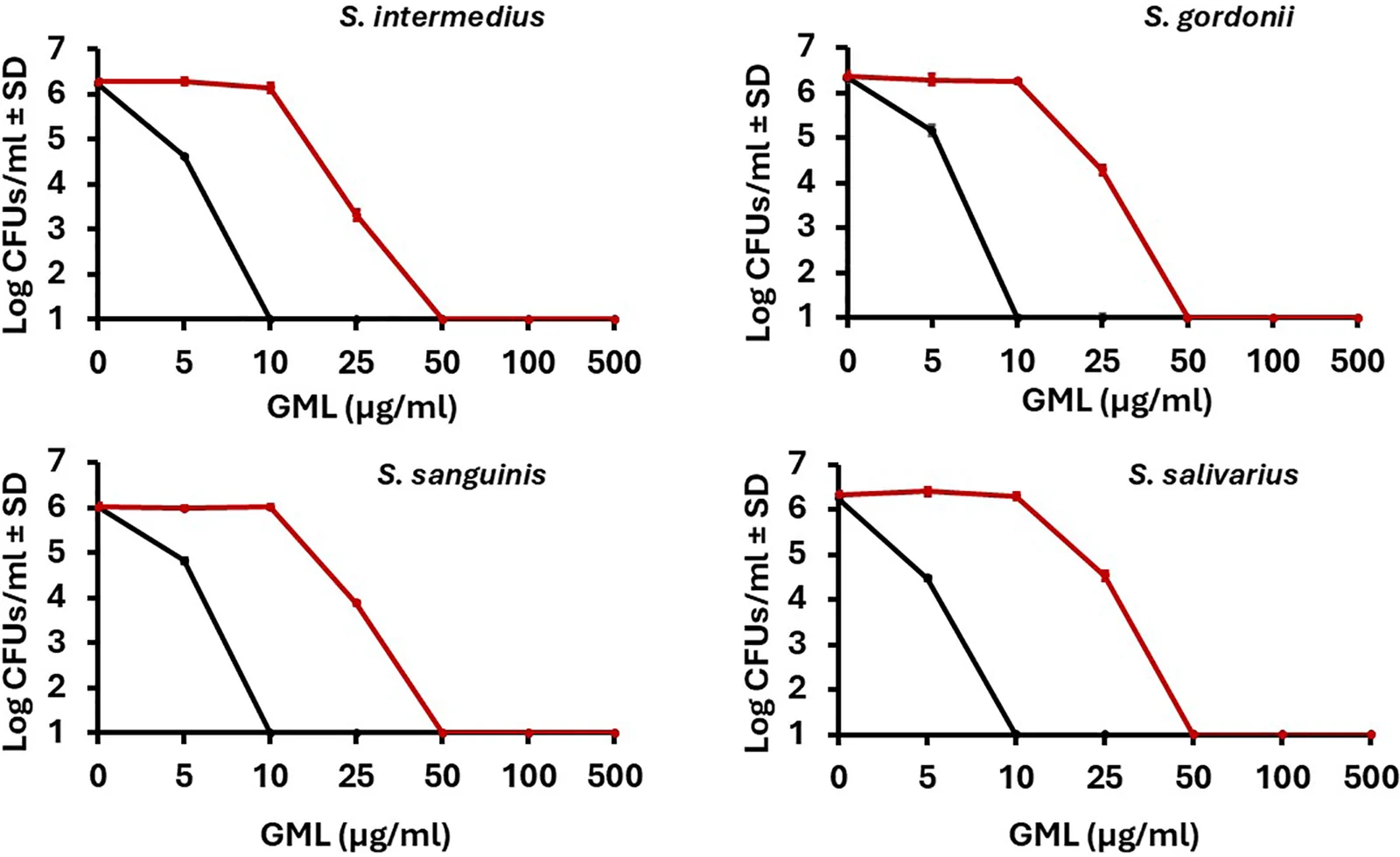
Effect of GML + petrolatum versus GML + PBS on four strains of viridans streptococci. Treatment groups were tested for 24 h at 37°C. Data are log_10_CFU/mL ± standard deviations (SD). Means as determined by Student’s *t*-test analyses were different between GML + petrolatum (black lines) and GML plus PBS (red lines) at *P* < 0.00001 for GML concentrations of 5, 10, and 25 µg/mL. Means at GML concentrations of 0, 100, and 500 µg/mL were not different.

Recently, it has been suggested that skin microbiome viridans streptococci may contribute to AD in very young children (21). Viridans streptococci include at least six subgroups of organisms commonly found in the throat and nose of all people but easily spread to the skin of very young children. We tested one viridans streptococcus from four common subgroups for susceptibility to GML in petrolatum versus GML in PBS (Fig. 7). It is important to emphasize that our experience with streptococci in general is that they do not produce lipases, unlike staphylococci. Consistent with that observation, we observed that all four viridans streptococci were killed by GML in petrolatum at concentrations of 10 µg/mL compared with 50 µg/mL for GML in PBS. Viridans streptococci were approximately 10-fold more susceptible to GML than staphylococci.

## DISCUSSION

The goal of this study was to assess whether glycerol monolaurate (GML) added to petrolatum could be used as a mild topical agent to prevent *S. aureus* and viridans streptococcal colonization of mucous membranes and skin to help in treatment of AD and diabetic ulcers. We showed that six *S*. *aureus* strains, both methicillin-resistant and methicillin-sensitive, were killed by GML in petrolatum, where the combination acted with enhanced activity. Importantly, all six *S*. *aureus* strains were completely killed by 500 µg/mL, with bactericidal activity (≥3 log reduction in colony-forming units in 24 h) at concentrations of GML of 50 and 100 µg/mL. We showed that GML has the greatest anti-staphylococcal activity compared with other related fatty acid monoesters of glycerol, all of which are generally recognized as safe by the Food and Drug Administration as food additives and for use in cosmetics.

Additionally, we showed that GML acted synergistically with petrolatum, compared with GML alone in PBS, to kill four viridans streptococcal strains. Viridans streptococci have recently been implicated in AD in very young children (21). As expected, GML killed streptococci at 10-fold lower concentrations than required to kill staphylococci. It is likely this difference results from the absence of lipase production by streptococci, compared with lipase production by staphylococci. Lipase production by *S. aureus* was shown to be the reason for the relatively high amount of GML required to kill the organisms (34). We did not evaluate the ability of GML in petrolatum to kill group A streptococci in the current study. These organisms have not been implicated in driving AD. However, we have previously shown that all group A streptococcal strains are highly susceptible (like viridans streptococci) to GML (3).

We have shown previously in humans that GML at 50,000 µg/mL, dissolved in K-Y Warming Gel, could be used to reduce colonization of the anterior nares by *S. aureus* and reduce vaginal *Candida albicans*, while at the same time increasing vaginal lactobacilli (3, 14). GML is soluble in petrolatum at 50,000 µg/mL, so there should be no expected problems with formulation of such a solution. In the prior clinical trial with GML solubilized in K-Y Warming Gel, several women noted transient stinging, presumably due to the non-aqueous K-Y Warming component (14). Women (vaginal) and adults (both male and female anterior nares) treated with GML in K-Y Warming Gel also noted the warming property of the mixture (6, 14). While this was not a problem for the study adult populations, it would potentially be a problem with treatment of the anterior nares of children and treatment of diabetic ulcers. We hypothesize that GML (10,000–50,000 µg/mL) in petrolatum would be a milder treatment.

In the current studies, we evaluated GML in petrolatum for killing four USA200 (CC30; TSST-1 positive), one USA300-like (SE-like X positive), and one USA400 (SEC positive) *S. aureus*. Previously, we studied possible reasons why skin and soft tissue infections associated with *S. aureus* are slow to heal. These studies are important both in management of atopic dermatitis and diabetes. The studies suggested that exotoxins, both superantigens and cytotoxins, delay wound healing (42, 43). Indeed, our studies showed that in many cases wounds will not heal at all unless TSST-1, present in the infections, are neutralized (43). Furthermore, the presence of TSST-1 in infection sites leads to more severe infections in atopic dermatitis and diabetes (25, 44, 45).

We also evaluated two coagulase-negative staphylococci for killing by GML in petrolatum, compared with GML in PBS. Some studies suggest that certain coagulase-negative staphylococci contribute to AD and diabetic ulcers (16, 24). Our study found that coagulase-negative strains are also killed by GML at 500 µg/mL, whether in petrolatum or PBS. Incidentally, we tested one additional *S. epidermidis* strain (data not presented), and this organism was killed completely by GML at 500 µg/mL. These may represent positive effects in AD and diabetic ulcer treatment. However, it could be hoped that normal flora microbes would not be killed. It has previously been shown that GML does not kill normal flora lactobacilli (14, 40). This effect was observed both *in vitro* (40), and in human studies where women were treated vaginally with 50,000 µg/mL of GML in K-Y Warming Gel (14). There was a 1.5 to 2 log increase in vaginal lactobacilli associated with the treatment (14). This positive effect of retaining normal vaginal lactobacilli likely resulted from those lactobacilli naturally producing an analog of GML called reutericyclin (3, 10). This is supported by both GML and reutericyclin being growth stimulants of lactobacilli, both having a broad and similar spectrum of antimicrobial activity, and both having a similar mechanism of action, that is dissipation of the potential difference across bacterial membranes (3, 10). This also suggests that addition of GML to non-aqueous gels, such as petrolatum and K-Y Warming Gel, would exhibit enhanced bactericidal activity because of disturbance of the membranes by both GML and the non-aqueous gels (3).

We previously showed that GML (50,000 µg/mL) in K-Y Warming Gel could kill *S. aureus* in a rabbit dermatitis model (39). We observed the same effect by 8 h post-treatment in the current study. Additionally, through *in vitro* studies, we showed that GML (500 µg/mL) in petrolatum was bactericidal to *S. aureus* by 1 h post-treatment with complete killing by 2 h.

There are multiple possible explanations for why GML in petrolatum is more antimicrobial than GML alone in PBS. Petrolatum is a complex mixture of lipids, which likely interact with bacterial plasma membranes, possibly increasing GML activity. Petrolatum clearly improves the solubility of GML, and this effect could increase the effective bioavailability of GML. Finally, the hydrophobic nature of petrolatum may promote prolonged contact with bacterial membranes, leading to the increased antimicrobial effect. As we showed previously, the non-aqueous gel, K-Y Warming, functions synergistically to kill bacteria (3). The same possible mechanisms of increased activity were proposed for K-Y Warming increasing activity.

In sum, we have shown that GML in petrolatum could be used, both *in vitro* and *in vivo*, to kill pathogenic staphylococci and potentially pathogenic streptococci. This included both methicillin-sensitive and methicillin-resistant *S. aureus*. Although GML belongs to a large class of fatty acid monoesters, all of which are generally recognized as safe by the FDA as additives to foods and cosmetics, among the group GML has the greatest anti-staphylococcal activity. This may have resulted because the lauric acid side chain in GML exactly spans one-half of the lipid bilayer. We suggest that future studies be done in humans to assess the *in vivo* efficacy of GML in preventing nasal colonization and topical treatment of AD and diabetic ulcers, the latter of which are notoriously resistant to treatment due to *S. aureus* colonization.

## MATERIALS AND METHODS

### Bacteria

*S. aureus* strains included the following. MN8 is a USA200 (CC30) menstrual toxic shock syndrome isolate (38). The strain is methicillin-sensitive, like most USA200 (CC30) strains, produces the superantigen TSST-1 and has a mutation in the α-cytotoxin gene, reducing the cytotoxin production by 50-fold (46). MNPE is a USA200 (CC30) menstrual TSS isolate from a fatal case of post-influenza hemorrhagic pneumonia (47). The strain is methicillin-sensitive, produces the superantigen TSST-1, and produces wild-type amounts of the cytotoxin α-toxin (*hla*; lacks the mutation that reduces the amount of the cytotoxin in >95% of menstrual TSS isolates). MNWH is a USA200 (CC30) menstrual TSS isolate from a recurrent case (48). The isolate is methicillin-resistant (has a hospital-associated SCC*mec* DNA element), produces TSST-1, and has the *hla* mutation, reducing the amount of the cytotoxin by 50-fold (48). Unusually, the MNWH strain lacks carotenoid pigment production that typifies *S. aureus*. Strain FRI1169 was originally obtained from Dr. Merlin Bergdoll (now deceased) from the University of Wisconsin Food Research Institute. This isolate was from a patient with menstrual TSS (49). The organism is USA200 (CC30), produces TSST-1, produces only low levels on α-toxin, and is methicillin-sensitive. MNKN is a USA400 (CC1) community-associated methicillin-resistant isolate from a fatal case of hemorrhagic pneumonia (50). The strain produces the superantigen staphylococcal enterotoxin C (SEC) and produces wild-type amounts of the cytotoxin α-toxin (50). HP1 is a recent clone of *S. aureus* from a patient with atopic dermatitis, and which is characterized by its hyper-pink growth on mannitol salt agar (30). The organism is related to USA300 (CC8), is methicillin-sensitive, and produces both SE-like X and wild-type α-toxin (30).

Strains of coagulase-negative staphylococci included a recent clinical isolate of *S. capitis* (41) and a normal microbiome culture of *S. epidermidis*. All organisms in this study are low passage and are maintained as −80°C frozen stocks in the Schlievert laboratory.

A *Lactobacillus crispatus* strain and four strains of viridans streptococci are maintained as −80°C frozen stocks in the Schlievert laboratory. The viridans streptococci included *Streptococcus intermedius*, *Streptococcus gordonii*, *Streptococccus sanguinis*, and *Streptococcus salivarius*.

### Fatty acid monoesters of glycerol

Glycerol monolaurate (GML) was purchased from Colonial Chemical Company, South Pittsburg, TN. The remaining compounds (glycerol monocaprate, glycerol monocaprylate, and glycerol monomyristate) were purchased from Sigma-Aldrich, St. Louis, MO. All compounds were solubilized in 100% ethanol at 100,000 µg/mL as stock solutions.

### *In vitro* experiments

All organisms for testing were first cultured overnight at 37°C with 200 rpm shaking in Todd Hewitt broth (Difco Laboratories, Detroit, MI) for staphylococci and stationary for lactobacilli and streptococci. Subsequently the appropriate dilutions were made in Todd Hewitt broth for inoculums of approximately 10^6^/mL or 10^7^/mL into Todd Hewitt broth, phosphate-buffered saline (0.005 M sodium phosphate, pH 7.2; 0.15M NaCl), or petrolatum (generic over-the-counter brand purchased from Walgreen’s Pharmacy).

In the first set of experiments, various glycerol monoesters (50 µL volumes in ethanol) individually were added to 25 mL volumes of Todd Hewitt broth in 125-mL Erlenmeyer flasks, with the corresponding control flasks having the same volumes of ethanol alone. Subsequently, 10^7^/mL of *S. aureus* MN8 were added to each flask. The flasks were then shaken (200 rpm) at 37°C for 24 h. Plate counts were then performed to determine CFUs/mL. It is important to note that glycerol monoesters at concentrations above 100 µg/mL in aqueous solutions are insoluble. However, the antimicrobial activity of these esters is increased above the solubility limit. This indicates that the monoesters likely embed into bacterial membranes, with more monoester becoming soluble, and the embedding process continuing until the bacteria die.

Petrolatum is a semi-solid at 37°C, and thus the compound was melted at 65°C and 2 mL added to sterile glass tubes. Then, the various tested concentrations of GML, PBS, or 100% ethanol (as control) in 20 µL volumes were added. Finally, the individual staphylococci were added to each tube in 20 µL volumes, and immediately the tubes were mixed and cooled to 37°C to prevent heat killing of the staphylococci, lactobacilli, and streptococci. The tubes were incubated for designated time periods, and plate counts were used to assess CFUs/mL.

Because GML in petrolatum and petrolatum alone are semi-solid are 37°C, we determined CFUs/mL by immersing sterile cotton swabs in the gels and plating directly with one swab onto sheep blood agar plates and then immersing a second swab into 0.9 mL of Todd Hewitt broth for additional serial dilutions in Todd Hewitt broths. Our past experience is that the swabs take up approximately 0.1 mL of volumes. Thus, the lowest dilution plated was the 10^−1^ dilution for a lower limit of detection of 10 CFUs/mL.

### Rabbit studies

Four New Zealand white rabbits (2–3 kg), both sexes, were used in these studies. The studies were performed with ABSL-2 conditions under an approved protocol by the University of Iowa IACUC (Number 3072547-001). Rabbits were administered buprenorphine for pain management by a University of Iowa veterinary technician. Then, the animals had both flanks shaved. The animals subsequently received approximately 10^9^ CFUs of *S. aureus* strain MN8 in 0.1 mL of PBS, painted onto the exposed skin over a 2 × 2 cm area. Then, the same areas were painted one-time with 0.5 mL of GML (50,000 µg/mL) in petrolatum or petrolatum alone. The animals were then returned to their cages and monitored for viable *S. aureus* at 8 h. For plate count determinations, the rabbits were swabbed diagonally across the infected area of exposed skin by rolling a PBS-wetted swab (holds 0.1 mL) one time. At 8 h, the veterinary technician euthanized the animals. Plate counts were performed on sheep blood agar plates after making dilutions in Todd Hewitt broths followed by immediate plating. Previously in similar experiments, we observed no toxicity to the rabbits of GML (39). Similar to that prior study, the animals remained healthy in appearance, had no unusual swelling or reddening of the flank application sites.

### Statistics

Data in graphs were presented as means ± standard deviations. Student’s *t*-test analysis was used to assess differences in mean values. All *in vitro* experiments were performed at least two times. The rabbit studies were performed one time only with *S. aureus* since our prior studies with GML in K-Y Warming Gel, and a more recent study with a compound related to GML, both showed killing of *S. aureus* (39, 51).

## Data Availability

Data and strains used in these studies are available upon request.

## References

[1] Li Q, Estes JD, Schlievert PM, Duan L, Brosnahan AJ, Southern PJ, Reilly CS, Peterson ML, Schultz-Darken N, Brunner KG, Nephew KR, Pambuccian S, Lifson JD, Carlis JV, Haase AT. 2009. Glycerol monolaurate prevents mucosal SIV transmission. Nature 458:1034–1038. doi:10.1038/nature07831 19262509 PMC2785041

[2] Haase AT, Rakasz E, Schultz-Darken N, Nephew K, Weisgrau KL, Reilly CS, Li Q, Southern PJ, Rothenberger M, Peterson ML, Schlievert PM. 2015. Glycerol monolaurate microbicide protection against repeat high-dose siv vaginal challenge. PLoS One 10:e0129465. doi:10.1371/journal.pone.0129465 26057743 PMC4461171

[3] Schlievert PM, Peterson ML. 2012. Glycerol monolaurate antibacterial activity in broth and biofilm cultures. PLoS One 7:e40350. doi:10.1371/journal.pone.0040350 22808139 PMC3394780

[4] Welch JL, Xiang J, Okeoma CM, Schlievert PM, Stapleton JT. 2020. Glycerol monolaurate, an analogue to a factor secreted by *Lactobacillus*, is virucidal against enveloped viruses, including HIV-1. mBio 11:e00686-20. doi:10.1128/mBio.00686-20 32371599 PMC7201201

[5] Strandberg KL, Peterson ML, Lin YC, Pack MC, Chase DJ, Schlievert PM. 2010. Glycerol monolaurate inhibits *Candida* and *Gardnerella vaginalis in vitro* and *in vivo* but not *Lactobacillus*. Antimicrob Agents Chemother 54:597–601. doi:10.1128/AAC.01151-09 20008774 PMC2812150

[6] Schlievert PM, Peterson ML. 2020. Decolonization of human anterior nares of *Staphylococcus aureus* with use of a glycerol monolaurate nonaqueous gel. mSphere 5:e00552-20. doi:10.1128/mSphere.00552-20 32727862 PMC7392545

[7] Kasrae H, Amiri Farahani L, Yousefi P. 2015. Efficacy of topical application of human breast milk on atopic eczema healing among infants: a randomized clinical trial. Int J Dermatol 54:966–971. doi:10.1111/ijd.12764 25640116

[8] Schlievert PM, Kilgore SH, Seo KS, Leung DYM. 2019. Glycerol monolaurate contributes to the antimicrobial and anti-inflammatory activity of human milk. Sci Rep 9:14550. doi:10.1038/s41598-019-51130-y 31601928 PMC6787265

[9] Upton EM, Schlievert PM, Zhang Y, Rauckhorst AJ, Taylor EB, Radoshevich L. 2023. Glycerol monolaurate inhibits *Francisella novicida* growth and is produced intracellularly in an ISG15-dependent manner. MicroPubl Biol. doi:10.17912/micropub.biology.000905 PMC10638595 37954520

[10] Lin XB, Lohans CT, Duar R, Zheng J, Vederas JC, Walter J, Ganzle M. 2015. Genetic determinants of reutericyclin biosynthesis in *Lactobacillus reuteri*. Appl Environ Microbiol 81:2032–2041. doi:10.1128/AEM.03691-14 25576609 PMC4345372

[11] Peterson ML, Schlievert PM. 2006. Glycerol monolaurate inhibits the effects of Gram-positive select agents on eukaryotic cells. Biochemistry 45:2387–2397. doi:10.1021/bi051992u 16475828 PMC2553893

[12] Witcher KJ, Novick RP, Schlievert PM. 1996. Modulation of immune cell proliferation by glycerol monolaurate. Clin Diagn Lab Immunol 3:10–13. doi:10.1128/cdli.3.1.10-13.1996 8770497 PMC170240

[13] Brosnahan AJ, Schlievert PM. 2011. Gram-positive bacterial superantigen outside-in signaling causes toxic shock syndrome. FEBS J 278:4649–4667. doi:10.1111/j.1742-4658.2011.08151.x 21535475 PMC3165073

[14] Mancuso AC, Widdice LE, Hughes BL, Schlievert P, Swamy GK, Stockdale CK, Bernstein DI, Winokur PL. 2020. Five percent monolaurin vaginal gel for the treatment of bacterial vaginosis: a randomized placebo-controlled trial. J Low Genit Tract Dis 24:277–283. doi:10.1097/LGT.0000000000000543 32379102

[15] Spaulding AR, Salgado-Pabón W, Kohler PL, Horswill AR, Leung DY, Schlievert PM. 2013. Staphylococcal and streptococcal superantigen exotoxins. Clin Microbiol Rev 26:422–447. doi:10.1128/CMR.00104-12 23824366 PMC3719495

[16] Simpson EL, Schlievert PM, Yoshida T, Lussier S, Boguniewicz M, Hata T, Fuxench Z, De Benedetto A, Ong PY, Ko J, Calatroni A, Rudman Spergel AK, Plaut M, Quataert SA, Kilgore SH, Peterson L, Gill AL, David G, Mosmann T, Gill SR, Leung DYM, Beck LA. 2023. Rapid reduction in *Staphylococcus aureus* in atopic dermatitis subjects following dupilumab treatment. J Allergy Clin Immunol 152:1179–1195. doi:10.1016/j.jaci.2023.05.026 37315812 PMC10716365

[17] Jeskey J, Kurien C, Blunk H, Sehmi K, Areti S, Nguyen D, Hostoffer R. 2024. Atopic dermatitis: a review of diagnosis and treatment. J Pediatr Pharmacol Ther 29:587–603. doi:10.5863/1551-6776-29.6.587 39659858 PMC11627575

[18] Leung DY. 2000. Atopic dermatitis: new insights and opportunities for therapeutic intervention. J Allergy Clin Immunol 105:860–876. doi:10.1067/mai.2000.106484 10808164

[19] Hill DA, Spergel JM. 2018. The atopic march: critical evidence and clinical relevance. Ann Allergy Asthma Immunol 120:131–137. doi:10.1016/j.anai.2017.10.037 29413336 PMC5806141

[20] Bantz SK, Zhu Z, Zheng T. 2014. The atopic march: progression from atopic dermatitis to allergic rhinitis and asthma. J Clin Cell Immunol 5:202. doi:10.4172/2155-9899.1000202 25419479 PMC4240310

[21] Shi B, Bangayan NJ, Curd E, Taylor PA, Gallo RL, Leung DYM, Li H. 2016. The skin microbiome is different in pediatric versus adult atopic dermatitis. J Allergy Clin Immunol 138:1233–1236. doi:10.1016/j.jaci.2016.04.053 27474122 PMC5235385

[22] Deng L, Costa F, Blake KJ, Choi S, Chandrabalan A, Yousuf MS, Shiers S, Dubreuil D, Vega-Mendoza D, Rolland C, Deraison C, Voisin T, Bagood MD, Wesemann L, Frey AM, Palumbo JS, Wainger BJ, Gallo RL, Leyva-Castillo JM, Vergnolle N, Price TJ, Ramachandran R, Horswill AR, Chiu IM. 2023. *S. aureus* drives itch and scratch-induced skin damage through a V8 protease-PAR1 axis. Cell 186:5375–5393. doi:10.1016/j.cell.2023.10.019 37995657 PMC10669764

[23] Nakamura Y, Oscherwitz J, Cease KB, Chan SM, Munoz-Planillo R, Hasegawa M, Villaruz AE, Cheung GYC, McGavin MJ, Travers JB, Otto M, Inohara N, Nunez G. 2013. *Staphylococcus* δ-toxin induces allergic skin disease by activating mast cells. Nature 503:397–401. doi:10.1038/nature12655 24172897 PMC4090780

[24] Vu BG, Stach CS, Salgado-Pabón W, Diekema DJ, Gardner SE, Schlievert PM. 2014. Superantigens of *Staphylococcus aureus* from patients with diabetic foot ulcers. J Infect Dis 210:1920–1927. doi:10.1093/infdis/jiu350 24951827 PMC4296179

[25] Vu BG, Stach CS, Kulhankova K, Salgado-Pabon W, Klingelhutz AJ, Schlievert PM. 2015. Chronic superantigen exposure induces systemic inflammation, elevated bloodstream endotoxin, and abnormal glucose tolerance in rabbits: possible role in diabetes. mBio 6:e02554. doi:10.1128/mBio.02554-14 25714716 PMC4358007

[26] Vu BG, Gourronc FA, Bernlohr DA, Schlievert PM, Klingelhutz AJ. 2013. Staphylococcal superantigens stimulate immortalized human adipocytes to produce chemokines. PLoS One 8:e77988. doi:10.1371/journal.pone.0077988 24205055 PMC3813495

[27] David MZ, Daum RS. 2010. Community-associated methicillin-resistant *Staphylococcus aureus*: epidemiology and clinical consequences of an emerging epidemic. Clin Microbiol Rev 23:616–687. doi:10.1128/CMR.00081-09 20610826 PMC2901661

[28] Moran GJ, Krishnadasan A, Gorwitz RJ, Fosheim GE, McDougal LK, Carey RB, Talan DA, Group EMINS. 2006. Methicillin-resistant *S. aureus* infections among patients in the emergency department. N Engl J Med 355:666–674. doi:10.1056/NEJMoa055356 16914702

[29] King MD, Humphrey BJ, Wang YF, Kourbatova EV, Ray SM, Blumberg HM. 2006. Emergence of community-acquired methicillin-resistant *Staphylococcus aureus* USA 300 clone as the predominant cause of skin and soft-tissue infections. Ann Intern Med 144:309–317. doi:10.7326/0003-4819-144-5-200603070-00005 16520471

[30] Schlievert PM, Kilgore SH, Yoshida T, Beck LA, Leung DYM. 2026. Observations on emergence of mannitol-use-deficient *Staphylococcus aureus*. Microbiol Spectr 14:e0309125. doi:10.1128/spectrum.03091-25 41416788 PMC12889070

[31] King JM, Kulhankova K, Stach CS, Vu BG, Salgado-Pabon W. 2016. Phenotypes and virulence among *Staphylococcus aureus* USA100, USA200, USA300, USA400, and USA600 clonal lineages. mSphere 1:e00071-16. doi:10.1128/mSphere.00071-16 27303750 PMC4899884

[32] Banke E, Rödström K, Ekelund M, Dalla-Riva J, Lagerstedt JO, Nilsson S, Degerman E, Lindkvist-Petersson K, Nilson B. 2014. Superantigen activates the gp130 receptor on adipocytes resulting in altered adipocyte metabolism. Metabolism 63:831–840. doi:10.1016/j.metabol.2014.03.004 24684823

[33] Armstrong DG, Tan TW, Boulton AJM, Bus SA. 2023. Diabetic foot ulcers: a review. JAMA 330:62–75. doi:10.1001/jama.2023.10578 37395769 PMC10723802

[34] Schlievert PM, Deringer JR, Kim MH, Projan SJ, Novick RP. 1992. Effect of glycerol monolaurate on bacterial growth and toxin production. Antimicrob Agents Chemother 36:626–631. doi:10.1128/AAC.36.3.626 1622174 PMC190568

[35] Projan SJ, Brown-Skrobot S, Schlievert PM, Vandenesch F, Novick RP. 1994. Glycerol monolaurate inhibits the production of beta-lactamase, toxic shock toxin-1, and other staphylococcal exoproteins by interfering with signal transduction. J Bacteriol 176:4204–4209. doi:10.1128/jb.176.14.4204-4209.1994 8021206 PMC205630

[36] Schlievert PM, Osterholm MT, Kelly JA, Nishimura RD. 1982. Toxin and enzyme characterization of *Staphylococcus aureus* isolates from patients with and without toxic shock syndrome. Ann Intern Med 96:937–940. doi:10.7326/0003-4819-96-6-937 7091971

[37] Lin YC, Schlievert PM, Anderson MJ, Fair CL, Schaefers MM, Muthyala R, Peterson ML. 2009. Glycerol monolaurate and dodecylglycerol effects on *Staphylococcus aureus* and toxic shock syndrome toxin-1 *in vitro* and *in vivo*. PLoS One 4:e7499. doi:10.1371/journal.pone.0007499 19838303 PMC2759527

[38] Schlievert PM, Kelly JA. 1984. Clindamycin-induced suppression of toxic-shock syndrome--associated exotoxin production. J Infect Dis 149:471. doi:10.1093/infdis/149.3.471 6715902

[39] Mueller EA, Schlievert PM. 2015. Non-aqueous glycerol monolaurate gel exhibits antibacterial and anti-biofilm activity against gram-positive and gram-negative pathogens. PLoS One 10:e0120280. doi:10.1371/journal.pone.0120280 25799455 PMC4370562

[40] Schlievert PM, Strandberg KL, Brosnahan AJ, Peterson ML, Pambuccian SE, Nephew KR, Brunner KG, Schultz-Darken NJ, Haase AT. 2008. Glycerol monolaurate does not alter rhesus macaque (*Macaca mulatta*) vaginal lactobacilli and is safe for chronic use. Antimicrob Agents Chemother 52:4448–4454. doi:10.1128/AAC.00989-08 18838587 PMC2592867

[41] Schlievert PM, Kilgore SH, Ford B, Leung DYM, Sekar P. 2025. Pro-inflammatory secreted virulence factors of *Staphylococcus capitis* causing a rare occurrence of severe native hip joint infection. ASM Case Rep 1:e00067-24. doi:10.1128/asmcr.00067-24 41244318 PMC12530238

[42] Merriman JA, Klingelhutz AJ, Diekema DJ, Leung DY, Schlievert PM. 2015. Novel *Staphylococcus aureus* secreted protein alters keratinocyte proliferation and elicits a proinflammatory response *in vitro* and *in vivo*. Biochemistry 54:4855–4862. doi:10.1021/acs.biochem.5b00523 26177220 PMC4912018

[43] Jacob HS, Vercellotti GM, Leung DYM, Schlievert PM. 2020. Case report of an unusual presentation of *Staphylococcus aureus* induced toxic shock syndrome/hyperimmunoglobulinemia E syndrome. Medicine (Baltimore) 99:e19746. doi:10.1097/MD.0000000000019746 32282735 PMC7220474

[44] Schlievert PM, Roller RJ, Kilgore SH, Villarreal M, Klingelhutz AJ, Leung DYM. 2021. Staphylococcal TSST-1 association with eczema herpeticum in humans. mSphere 6:e0060821. doi:10.1128/mSphere.00608-21 34319127 PMC8386428

[45] Woods B, Kilgore SH, Nikiciuk M, Ripley B, Upton EM, Radoshevich L, Phipatanakul W, Leung DYM, Geha RS, Schlievert PM. 2025. Skin levels of toxic shock syndrome toxin-1 predict the severity of atopic dermatitis. J Allergy Clin Immunol 156:480–482. doi:10.1016/j.jaci.2025.05.018 40526056 PMC12925615

[46] Lin YC, Anderson MJ, Kohler PL, Strandberg KL, Olson ME, Horswill AR, Schlievert PM, Peterson ML. 2011. Proinflammatory exoprotein characterization of toxic shock syndrome *Staphylococcus aureus*. Biochemistry 50:7157–7167. doi:10.1021/bi200435n 21749039 PMC3156861

[47] MacDonald KL, Osterholm MT, Hedberg CW, Schrock CG, Peterson GF, Jentzen JM, Leonard SA, Schlievert PM. 1987. Toxic shock syndrome. a newly recognized complication of influenza and influenzalike illness. JAMA 257:1053–1058. doi:10.1001/jama.257.8.1053 3806893

[48] Schlievert PM, Strandberg KL, Lin YC, Peterson ML, Leung DY. 2010. Secreted virulence factor comparison between methicillin-resistant and methicillin-sensitive *Staphylococcus aureus*, and its relevance to atopic dermatitis. J Allergy Clin Immunol 125:39–49. doi:10.1016/j.jaci.2009.10.039 20109735 PMC2814367

[49] Bergdoll MS, Crass BA, Reiser RF, Robbins RN, Davis JP. 1981. A new staphylococcal enterotoxin, enterotoxin F, associated with toxic-shock-syndrome *Staphylococcus aureus* isolates. Lancet 1:1017–1021. doi:10.1016/s0140-6736(81)92186-3 6112412

[50] Kravitz GR, Dries DJ, Peterson ML, Schlievert PM. 2005. Purpura fulminans due to *Staphylococcus aureus*. Clin Infect Dis 40:941–947. doi:10.1086/428573 15824983

[51] Schlievert PM, Brennan PE, Klem RE, Reardan DT. 2025. Antifungal, antibacterial, and anti-inflammatory activity of glycerol dithionomonolaurate, an analog of glycerol monolaurate. mSphere 10:e0031825. doi:10.1128/msphere.00318-25 41036846 PMC12570472

